# Combining Structural Modeling with Ensemble Machine Learning to Accurately Predict Protein Fold Stability and Binding Affinity Effects upon Mutation

**DOI:** 10.1371/journal.pone.0107353

**Published:** 2014-09-22

**Authors:** Niklas Berliner, Joan Teyra, Recep Çolak, Sebastian Garcia Lopez, Philip M. Kim

**Affiliations:** 1 Terrence Donnelly Centre for Cellular and Biomolecular Research (CCBR), University of Toronto, Toronto, Ontario, Canada; 2 Department of Molecular Genetics, University of Toronto, Toronto, Ontario, Canada; 3 Department of Computer Science, University of Toronto, Toronto, Ontario, Canada; 4 Universidad Nacional de Colombia, Manizales, Colombia; University of Alberta, Canada

## Abstract

Advances in sequencing have led to a rapid accumulation of mutations, some of which are associated with diseases. However, to draw mechanistic conclusions, a biochemical understanding of these mutations is necessary. For coding mutations, accurate prediction of significant changes in either the stability of proteins or their affinity to their binding partners is required. Traditional methods have used semi-empirical force fields, while newer methods employ machine learning of sequence and structural features. Here, we show how combining both of these approaches leads to a marked boost in accuracy. We introduce ELASPIC, a novel ensemble machine learning approach that is able to predict stability effects upon mutation in both, domain cores and domain-domain interfaces. We combine semi-empirical energy terms, sequence conservation, and a wide variety of molecular details with a Stochastic Gradient Boosting of Decision Trees (SGB-DT) algorithm. The accuracy of our predictions surpasses existing methods by a considerable margin, achieving correlation coefficients of 0.77 for stability, and 0.75 for affinity predictions. Notably, we integrated homology modeling to enable proteome-wide prediction and show that accurate prediction on modeled structures is possible. Lastly, ELASPIC showed significant differences between various types of disease-associated mutations, as well as between disease and common neutral mutations. Unlike pure sequence-based prediction methods that try to predict phenotypic effects of mutations, our predictions unravel the molecular details governing the protein instability, and help us better understand the molecular causes of diseases.

## Introduction

Any two human genomes differ in a number of different ways. There are changes on the level of individual nucleotides (Single Nucleotide Polymorphisms – SNPs or Single Nucleotide Variants – SNVs, depending on frequency) as well as many larger ones, such as deletions, insertions, and copy number variations. Any of these may cause alterations in an individual's phenotype, with effect size ranging from near neutral to very strong. In addition, many complex diseases, such as cancer, are caused by somatic mutations (i.e., are acquired during the individual's life). In general, SNPs can occur either at non-coding [1] or at coding regions [2], in which only non-synonymous SNPs (nsSNPs) induce a change in the amino acid sequence and, generally, have stronger effects on the phenotype. Identifying those nsSNPs that infer susceptibility or protection to complex diseases will aid early diagnosis, prevention and treatments [3]. Recent efforts to map human genetic variation by sequencing exomes [4] and whole genomes [5]–[7] have characterized the vast majority of common and low frequency SNPs and many structural variants. In total, about 64 million human SNPs have already been discovered and genotyped [8]. In addition, comprehensive catalogues of somatic mutations from different human cancer genomes are being made in order to understand the primary causes of cancer [9], [10]. Yet, experimental characterization of the effects of every single mutation is virtually impossible due to the time, cost and difficulty [3], [11].

The existence of these databases combined with available biochemical data in single-point mutations on protein domains [12] and complexes [13] prompted the development of automatic computational methods able to predict the effect of mutations accurately. These tools are based on either sequence, structure or energy features, either in isolation or in combination using machine learning approaches [14]. Most of the sequence-based methods rely on evolutionary conservation of homologous protein sequences, since functional regions are known to be conserved [15]. The most popular method among the sequence-based algorithms is SIFT (Sorting Intolerant from Tolerant) [16], which uses multiple sequence alignments of homologous sequences to generate scores at each position based on patterns of amino acid substitutions. However, they can't provide molecular details that can help understand the mechanism ultimately causing the disease. As an alternative to increase accuracy, some methods have complemented sequence features with structural ones in globular domains incorporating parameters such as stability, solvent accessibility and domain boundaries among others. In order to extract the structural features, some methods map the sequence to the closest 3D protein structure (if it exists) [17], whereas others build homology models based on the closest structure to increase accuracy [18]. All these methods are trained using mutations with known phenotypes (e.g., from diseases) in order to predict the effect of mutations with unknown phenotypes at proteome scale.

Other methods predict free energy differences between wild type and mutant variant by training in limited experimental dataset, and while not perfect, are usually able to obtain a correct trend [19]. The most accurate physical methods such as molecular dynamics or Monte Carlo simulations, are computationally very demanding, and hence, not applicable to large datasets [20]–[22]. In contrast, empirical potential approaches provide a fast and quantitative alternative to estimate the contribution of a substitution to the stability of proteins [23]–[25]. These approaches incorporate physical and statistical energy terms that are weighted to fit experimental data, and have been proven to be qualitatively accurate, although fail to provide precise values [19]. The most successful methods to predict the effect of mutations in protein cores use a combination of some of the above (i.e. sequence, structural and energetic features) [24], [26], [27] by applying machine learning methods, e.g., neural networks, support vector machines (SVMs), or random forests. Such methods leverage information about mutations to fit a non-linear function to experimental data on protein stability changes upon single-point mutations [28]–[30]. Recently, a stability meta-predictor for core mutations was shown to achieve remarkable performance by integrating the output of several prediction tools [31].

With current approaches, mutations at the surface of a domain are usually considered neutral. In globular domains, the effect of each mutation is determined by its structural context; whereas mutations in the core may alter the stability of the domain fold, mutations at the surface regions that are involved in molecular recognition may directly affect binding affinities. In fact, many, if not most, disease-related mutations are located at the interface of protein complexes [32], [33]. A small subset of interface residues, called hot spots, are critical for complex formation and their identification is of major importance. Computational alanine scanning methods based on empirical energy functions have been extensively used to identify all relevant positions for complex formation [34]. For the first time, these methods have been evaluated in predicting the effects of multiple amino acid mutations in new experimental data generated for single point mutation variants of a given protein-protein interaction [35]. Their results confirm a weak performance of the current methods, in which machine learning techniques show the best results. A novel energy-based approach specifically trained for the prediction of mutational effects in protein complexes has shown relatively good results, although its performance was not comparable to the methods evaluating core mutations [36]. This is presumably due to the biophysical nature of protein-protein interfaces, where properties more difficult to estimate, such as polar and electrostatic terms, play a much more important role than in the domain core [35]. Instead of predicting differences in binding free energies, Agius and colleagues estimate the dissociation rates upon residue mutation omitting association rates [37]. This is done by performing computational alanine scans of the interfacial residues pre- and post- mutation in order to capture synergistic effects that help them to relate the changes in energetics to dissociation rates upon mutation. Their best machine learning methods achieve a correlation of 0.79 with experimental off-rates for the prediction of stabilized mutants [37].

Energy-based predictive tools have never been exploited at proteome scale, despite providing more accurate predictions than sequence-based tools and also giving molecular insight into the effect of the mutation [38]. Their applicability has been limited by the extensive computational resources required and by the relatively low structural coverage of the proteome [39]. Interestingly, recent efforts have shown that the structures of many human proteins and their complexes can be modelled using structural templates of the PDB repository in a large scale, even at low similarity between the homolog template and target sequences [39]. Other studies have also shown that stability predictions in structural models created with templates at different similarities do not lose predictive power compared to the experimentally solved structures [40]. An interesting study has recently analyzed the effects of glioblastoma missense mutations affecting protein complexes by modelling the structure of the complexes with a mutation in the interface, and by evaluating the stability effect using an empirical potential approach [41]. These results highlight the importance of these strategies to understand the molecular mechanisms of disease mutations, but their limitations also underline the requirement of integrative machine learning approaches to improve predictive accuracy.

Here, we developed a novel Ensemble Learning Approach for Stability Prediction of Interface and Core mutations (ELASPIC). The framework boosts predictive power by integrating a wide range of sequence and structural features using the Stochastic Gradient Boosting of Decision Trees (SGB-DT) algorithm. Using high quality experimental datasets, our results show that ELASPIC outperforms all other methods in predicting the effect of core, and specifically, interface mutations. We also integrate our predictor with homology modeling and demonstrate the accuracy of our predictor when used on modeled structures, showing that our predictor can be used at a proteome-wide scale. Finally, we show that it correctly distinguishes harmless from diseases-associated genetic mutations. Therefore, our work opens new perspectives in genome-wide identification of disease-causing mutations, not only in predicting stability changes in protein cores and interfaces, but in rationalizing the molecular principles behind such mutations.

## Results/Discussion

**Figure 1:**
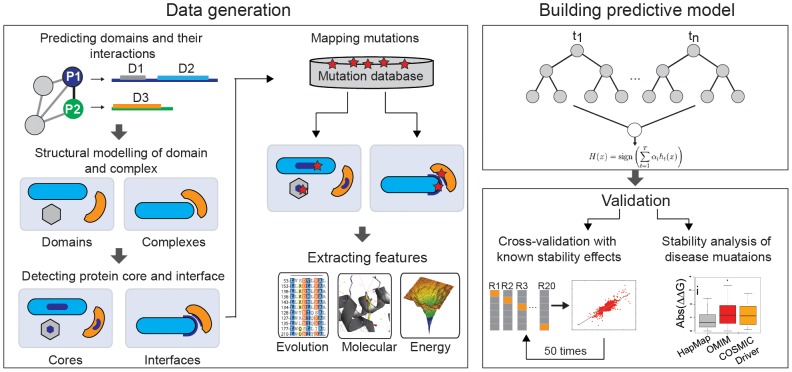
ELASPIC methodology. Schematic view of the strategy used to derive predictive features and train and validate ELASPIC for the prediction of stability effects in domain core and domain-domain interfaces upon mutation.

We developed ELASPIC, a method to predict stability effects induced by mutations in the core of a domain and in the interface of a complex. First, we extracted a set of sequence, molecular and energetic features from the two training sets containing biochemical data for wild type and mutant variant (Figure 1). We use the wild type structure of the domain/complex and the modelled mutant variant to extract the non-sequence features. In the model building phase, we use a Stochastic Gradient Boosting of Decision Trees (SGB-DT) algorithm to fit a non-linear function to minimize the prediction error, and perform a comprehensive evaluation of ELASPIC performance on predicting the effect of mutations in core and interface of proteins, using both experimental and homology-based modelled structures. Finally, we run ELASPIC at proteome scale by modelling domains and domain-domain complexes in a large-scale (Figure 1), and evaluate performance on both published benchmark datasets as well as on newly obtained disease related and HapMap mutation datasets.

### Benchmark competition of core mutations on experimental structures

**Figure 2:**
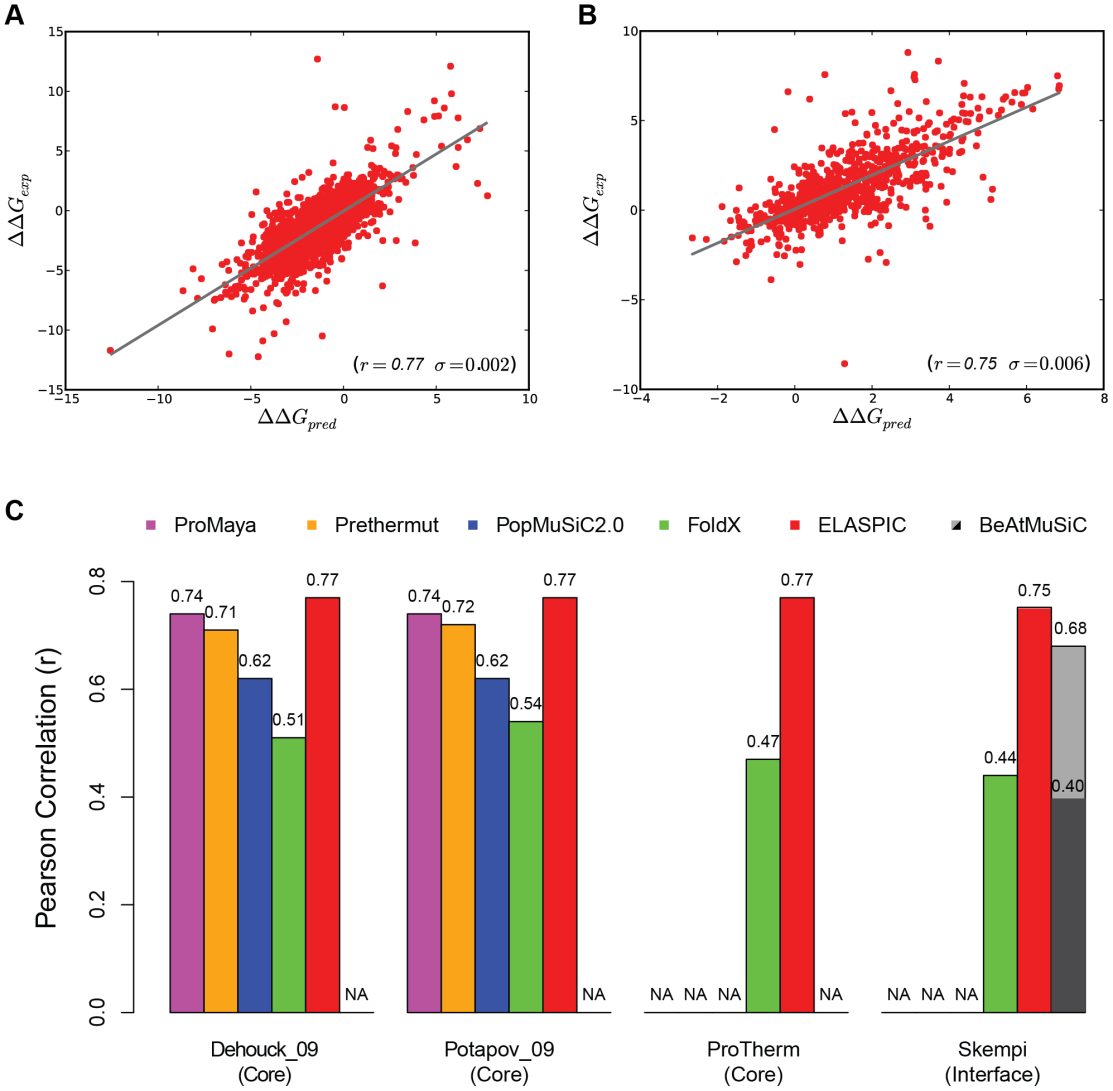
Summary of the results. Correlation between predicted and experimental ΔΔG values for our curated ProTherm core dataset (A) and SKEMPI interface dataset (B). (C) Comparative histograms of the Pearson correlation among several state-of-the-art methods using three versions of ProTherm datasets for the core predictions, and SKEMPI dataset for the interface prediction.

**Figure 3:**
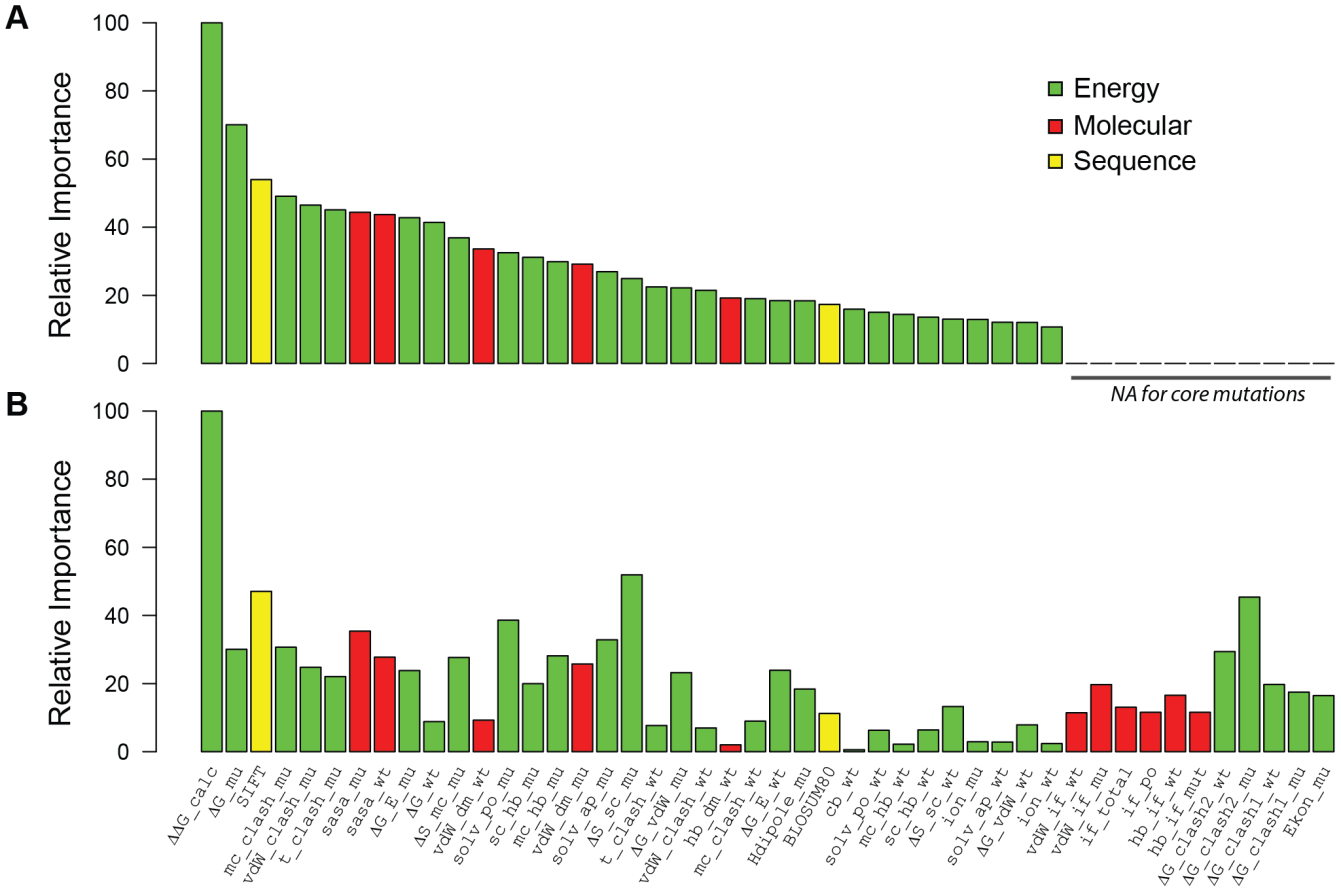
Feature importance for core and interface predictions. Histogram representing the relative importance of the different features for core predictions (A) and interface prediction (B). To avoid cluttering, only features with a relative importance of 10% or larger were considered and coloured according to the three categories. Abbreviations: t: torsional, diS: disulfide, E: electrostatics, ion: ionization, dS: entropy, Hdipole: helix dipole, cb: covalent bond, sb: salt bridge, hb: hydrogen bond, cisb: cysteine bond, wb: water bridge, vdW: wan der Waals, mc: main chain, sc: side chain, if: interface, dm: domain, sasa: solvent accessibility, solv: solvation, ap: apolar, po: polar (see Table S1 for feature description).

To train and asses our model in predicting stability effects of core mutations, we used ProTherm [12], the most extensive database of experimentally measured stabilities for protein domains with known structure. To avoid over fitting in our predictions, we created an unbiased, non-redundant, high-quality training set of 3,463 domains and their mutant variants, and extracted the sequence, structure and energy features (Figure 1, Table S1). The predictive power was assessed by a rigorous 20-fold cross-validation procedure. Our training results show a Pearson correlation coefficient (r) of 0.77 (standard deviation, σ = 0.002 and root mean square error, RMSE = 1.20) between experimental and predicted ΔΔG values (Figure 2A). Interestingly, the correlations only differ by 0.01% from training on full ProTherm dataset (r = 0.78), pointing out a low redundancy of ProTherm dataset. To gain a more comprehensive understanding of the importance of single features in fitting the experimental data, we analyzed the predictive power of each feature relative to the most predictive one as inferred by the SGB-DT's built-in feature importance calculation method (Figure 3A and Methods). The two most predictive features were the free energy difference between wild type and mutant, ΔΔG_fold_, and the ΔG_mutant_, calculated by FoldX. Interestingly, sequence conservation based on scores from SIFT are the third most informative feature in assessing the stability change. This result is consistent with the view that residues important for the domain fold will also show high evolutionary conservation [42]. In addition, the molecular features describing volumetric features of the wild type and mutant residues, such as surface accessibility or van der Waals distances, show special relevance, as previously described [43] (Figure 3A).

Many methods to predict stability effects of mutations have been developed [14]. Unfortunately, a direct comparative analysis of the methods is unfeasible [18]; different ProTherm [12] versions and filtering methodologies have been used by the different authors. In addition, most of these methods are only available as web servers for small-scale prediction purposes, but not for training and evaluation in batch mode, which is required for a proper comparative experiment [18]. To make a comparison as unbiased as possible with our method, we selected two sub-set datasets that were widely used as benchmark in multiple comparative studies: Potapov_09 [19] and Dehouck_09 [24]. We trained and validated ELASPIC individually and independently for each dataset by a 20-fold cross validation procedure to compare our performance with different methods: an energy-based method (Prethermut [44]), a statistical method using sequence and structural descriptors (PopMuSiC2.0 [24]), and a random forest-based method combining the features of the two previous methods (ProMaya [31]). ELASPIC consistently shows better performance than previous methods evaluated on the same datasets showing a consistent r of 0.77 (Figure 2C). However, the performance increase is not large in the stability case, suggesting that previous methods are already highly optimized, and that we are close to the limit imposed by the intrinsic errors in experimental methods to calculate protein stability. FoldX shows a loss of performance compared to its original publication [23]. This behaviour has already been observed in a previous comparative study [19], and could be attributed to the different dataset used and the parameters for the energy function that were calibrated a decade ago for a small dataset. As expected, Prethermut does better than FoldX, since it is a machine learning approach based on FoldX energy terms. Pro-Maya uses its own sequence- and molecular-based features in combination with the ΔΔG_fold_ scores from Prethermut and the statistical potentials based score from PoPMuSiC-2.0. As expected, ProMaya shows a better performance compared to Prethermut and PopMuSiC2.0 independently (0.74 vs 0.71 and 0.62, respectively). We attribute ELASPIC's improvement to two factors. First, we found that SGB-DT gave slightly better performance (∼0.02 higher) compared to the Random Forest algorithm, which is the one used in ProMaya. We presume that the number and variety of features might help our method to better balance the different features in order to fit the experimental values, as observed in Figure 3A. The second factor is that ProMaya relies on a single ΔΔG prediction value from Prethermut, whereas we provide all independent FoldX energy terms as features in combination with molecular and sequence features.

### Benchmark competition of interface mutations on experimental structures

Many disease-related mutations have been found at the interface of protein complexes [32], [33]. These mutations can disrupt protein-protein interactions, affecting signalling pathways and leading to diseases. Most current prediction methods are almost entirely focused on protein cores, and can only predict the effect of surface mutations as neutral or near-neutral, including interface mutations. This lack of methods is due to a number of factors, including to the relatively low coverage of existing structures of protein interactions. Analogous to the core case, features are extracted from experimental structures of a complex and its mutant model (Figure 1). The interface method includes a set of extra molecular and energetic features describing the interface of the complex in detail (Table S1). The FoldX energy terms are obtained from the calculation of the binding free energy (ΔΔG_bind_). To train and asses our model in predicting stability effects of interface mutations, we obtained the training set from the SKEMPI [13] database, the most abundant resource of experimental ΔΔG of mutations in the interface of protein-protein complexes. As for the core mutations, the predictive power was assessed by a rigorous 20-fold cross-validation procedure (see Methods). Our results showed a correlation coefficient of 0.81 (σ = 0.003), significantly outperforming current methods. Accurate analysis of the SKEMPI data revealed a high redundancy of complexes and mutations, suggesting a biased and over-estimated performance of the model. We corrected the problem by applying a 90% sequence identity redundancy reduction step and obtained 873 out of 2,045 instances (see Methods). For machine learning based approaches that rely on cross-validation for hyper-parameter optimization and performance assessment, this reduction is crucial and performance would be mistakenly over estimated if redundancy is not eliminated. Note that the problem is less severe in the core dataset with less redundancy (∼30%) than in the interface dataset (redundancy ∼60%) as also revealed by the small difference in performance of pre- and post- redundancy removal cross validation experiments.

With the curated dataset, our results decreased to a correlation coefficient of 0.75 (σ = 0.006, RMSE = 1.26), which is still comparable in accuracy to the predictions of the core predictive model (Figure 2C). A recently developed coarse-grained predictor, BeAtMuSiC [36], reports a correlation of 0.40 on their SKEMPI curated set, and 0.68 after removing 10% outliers. In our modified dataset, we obtain r = 0.75, whereas BeatMuSiC obtains only 0.53 (Figure 2C). This significant improvement can be attributed to both the inclusion of a large and diverse set of features and our supervised machine learning approach. In this regard, the feature importance analysis revealed as essential the role of ΔΔG_bind_ for the interface prediction as it was ΔΔG_fold_ for core. Other energy terms such as sidechain entropy, energy clashes and solvation of mutant residues become also relevant. Sidechain entropy and solvation might differentiate between mutations at the center or rim of the interface, whereas energy clashes might indicate impossible amino acid substitutions in the center of the interface due to the size constraint. As for core, SIFT scores are of major importance, suggesting a lower tolerance to mutation of highly conserved residues in interfaces [45]. Many molecular features play a minor role in the prediction of stability changes, including most of the interface descriptors.

Prediction of the impact of a mutation on protein interfaces is more challenging than on the protein core for several reasons. The first one is the diversity of physicochemical properties of interfaces (i.e., sizes and amino acid composition); some interfaces are wide, flat and hydrophobic resembling protein cores, whereas others are small and hydrophilic [46], [47]. Second, the specific nature of many interactions may be difficult to render accurately by the FoldX energy function. Finally, it is more difficult to model the mutant side-chain conformation since interfaces accept a greater variety of residue changes [48], whereas good side-chain modelling is critical for a good ΔΔG estimation. This is reflected on our FoldX results, where we obtained a correlation coeficient of 0.54 for the core predictions and 0.44 for the interface. To the best of our knowledge ELASPIC is the first integrative approach that uses multiple features to predict the stability changes of point mutations in the interface of protein-protein complexes. The results are comparable to our stability predictor for core mutations, suggesting the suitability of the features to describe changes to protein stability as well as to estimate binding affinities despite the distinctive physicochemical principles governing both molecular processes.

### Quality assessment of predictions based on experimental vs. modelled structures

While the coverage of the PDB of any given proteome is low, it can be greatly expanded by modeling proteins and their complexes using homologous structures as templates [39]. Hence, we integrated a homology modeling component to generate structural models for ProTherm and SKEMPI datasets, and evaluated the potential loss in accuracy derived from the usage of imperfect structural models to extract molecular and energetic features. In order to maximize the amount of data and avoid biases, we trained our predictor independently for each dataset by leave-one-family-out cross validation to optimize the procedure (See Methods for details). The modeled ProTherm dataset contains ∼40% of the models done with structural templates below 90% sequence similarity. However, obtaining structural templates of protein-protein complexes was more difficult to achieve for SKEMPI, and our modeled dataset contained template similarity above 90% for ∼80% of the cases. The generation of these modeled datasets, specially for complexes, will improve with time by the increase of a much more diverse set of solved structures of protein complexes in the PDB repository.

Our training results show a correlation coefficient of 0.52 and 0.4 for ProTherm and SKEMPI, respectively (Figure S1 and S2). As expected, the results using modeled structures are lower than the correlations obtained using experimental structures (0.77 and 0.75), yet we still significantly improve the correlations obtained by using FoldX alone (0.45 and 0.27). In addition, we observe that modelling introduces systematic biases in the features (Figure S3 and S4), although the feature importance remains similar between experimental and modelling-based training (Table S3). This may be an inherent weakness in current homology modeling techniques that we sidestep by building and retraining a separate predictor. For the nine most important features for core and interface mutations, the box plot distribution shows that features extracted from modeled structures have a better agreement with the disease mutation features (Figure S3 and S4). In addition, the figures show that the range and the values of the features from diverse methods are not suitable for training the model on one type of data and using it on another. This result stresses the importance of training on modeled structures in order to predict effects of mutations proteome-wide, in which approx. 80% of the human proteome has to be modelled [39]. We rely on the potential of the algorithm to learn from modelling and energy calculation biases introduced by the different methods to calibrate predictions. Yet, the overlap between distributions should improve in the future by using much larger datasets of stability and binding experimental measurements, without the requirement of having an experimental structure, as long as the proteins and their complexes can be modelled by homology.

### Stability prediction in disease associated mutations

In order to evaluate ELASPIC performance in identifying significant differences between harmless and disease-related mutations genome-wide, we created four mutation categories with distinct phenotypic signatures. First, we collected a set of non-synonymous SNPs from HapMap, which are thought to be neutral or near-neutral. Second, we used mutations from OMIM, which are high-penetrance mutations that cause congenital diseases and follow Mendelian inheritance. As such, these are expected to have a relatively strong effect. Finally, we collected somatic cancer mutations from COSMIC that we split into two groups: “driver” mutations defined as causal mutations that lead to abnormal growth and tumor formation, and “passenger” mutations, which are results of a breakdown of the DNA repair mechanisms and accumulate more or less at random.

We developed a methodology to map any mutation in the proteome to a structural domain, and accurately distinguish whether the mutation falls in the core, a binding region or another surface region of the domain (Figure 1 and Methods section). The approach combines Pfam domain detection with boundary extension using the closest homolog of known structure. The templates provide us with the core information and allows us to model the structure of the domain. To identify binding regions for a given protein domain, we check its partners in a protein-protein interaction network and their domain composition (Figure 1; see Methods). Only those domain-domain combinations between proteins with an existing complex of known structure are selected for further analysis. As for single domains, the closest homologous template provides us with the interacting positions and allows us to model the structure of the complex. Once a mutation is found either in the core or interface, the modeled structure is used to extract the respective features required for prediction. Only mutation results showing ΔG_wt_ ≤30 and relative DOPE score <1 were accepted for comparative analysis (see Methods). These cut-offs were obtained from the analysis of the modelled training set, and should identify and eliminate aberrant models, non-folding domains and non-interacting domain pairs. Finally, we predicted the ΔΔG_DT_ (i.e., the predicted ΔΔG based on our SGB-DT model) for each mutation using our regression model that represents in kcal/mol the stability of the domain fold for core mutations, and the stability of the domain-domain complex for the interface mutation.

**Figure 4:**
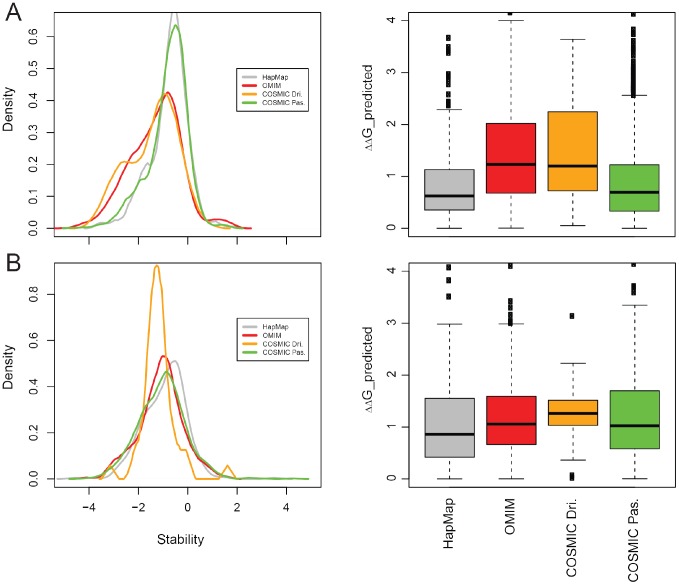
Summary of stability prediction of nsSNP mutations. Predicted absolute ΔΔG_DT_ box plots (right) are shown for (A) core and (B) interface mutations and the three types of mutations (Hapmap, OMIM and COSMIC driver/passenger).

In total, we obtained 2,637 mutations in domain cores, and 1,383 mutations in domain-domain interfaces distributed among the different disease groups (Table 1). As shown (Table S4), our ΔΔG_DT_ predictions for core mutations show a statistically significant difference in the box plot distributions between groups (Figure 4A and Table S4). HapMap SNPs show the lowest median and, on average, only lead to a slight destabilization of the proteins that harbour them, consistent with the fact that they are likely only under weak purifying selection. Likewise, COSMIC passenger mutations have a low median, again consistent with the fact that these are random somatic mutations and do not undergo selection; yet many outliers showing strong effects are observed, pointing out that the border distinguishing driver from passenger mutations based on occurrence of the mutation is not optimal. As expected, OMIM SNPs and COSMIC driver mutations show much stronger effects at the core of protein domains than HAPMAP and COSMIC passengers due to their direct link to genetic disorders and cancer, respectively. Our results suggest that in many cases the phenotypic effect of the mutation might be caused by the destabilization of the domain core, which might affect the correct protein function. A difference of 2 kcal/mol that we observe for these two disease related mutations is considered to truly destabilize cores of proteins, something that has already been observed to happen in OMIM disorders [49]. A small number of disease mutations produce over-stabilization of domain cores that show no differences between groups (Figure 4).

**Table 1:** Disease mutations calculated for domain cores and interfaces.

Database	Core Mutations	Interface Mutations
HAPMAP	415	310
OMIM	700	291
COSMIC passenger	1445	736
COSMIC driver	77	48

Our ΔΔG_DT_ predictions for interface mutations show differences in the box plot distributions between groups similar to the observations made for the core distributions. Most of the COSMIC driver mutations have binding energy differences around 1.2 kcal/mol, while the other three groups have lower values. Mutations destabilizing protein complexes should have a ΔΔG comparable to the contribution of a hot spot residue to binding which is considered to exceed 1.5 kcal/mol [50], [51]. It is important to note that both HapMap and COSMIC passenger mutations show similar distributions and contain mutations that severely affect binding affinity, suggesting that many disruptions of interactions may not lead to strong phenotypic consequences (Figure 4B and Table S4). However, the phenotypically more severe OMIM and COSMIC driver mutations have a higher fraction of interaction disrupting mutations. This suggests that many of these diseases might be caused by disruptions of protein-protein interactions that might affect the correct cellular function, as suggested by the concept of ‘edgetics’ [52]. According to our results there is only a limited number of mutations that over-stablize protein complexes, especially for COSMIC drivers (Figure 4B). Unfortunately, due to the small size of some datasets, several differences between groups are not statistically significant (Table S4). Nevertheless, we are already able to observe the expected trend between disease classes that is also shown for core mutation effects (Figure 4B). We expect this trend to improve in the future with increasing number and diversity of structures of protein complexes in the PDB that could facilitate homology modelling.

### Conclusions

The human population is estimated to have 67,000–200,000 common nsSNPs [53], [54], and each person is thought to be heterozygous for 24,000–40,000 nsSNP [53]. Experimental characterization of the impact of each nsSNP on protein function is unfeasible in terms of time, cost and difficulty. In addition, the computational prediction of effects of mutations in the proteome at high accuracy using energy based models is extremely challenging [14]. Based on the assumption that mutations affecting protein function tend to occur at evolutionary conserved regions, such as active sites or in protein cores, current methods are based on purely sequence features, such as SIFT [16]. Other methods combined sequence with molecular features obtained from homologous sequences, such as PolyPhen-2 [17], in order to assess the effect of the mutation in the stability of the protein. Despite the limitations of these techniques [17], there is no integrative predictor including energetic features that has been tested in a large scale.

We introduce ELASPIC, a novel ensemble learning approach that is able to predict stability effects upon mutation in both domain cores and domain-domain interfaces. We combine for the first time semi-empirical energy terms, sequence conservation, and a wide variety of molecular details with a Stochastic Gradient Boosting of Decision Trees (SGB-DT) algorithm. Our 75 features are mainly based on a molecular description of the mutated position, the energy terms describing the free energy difference between wild type and mutant variant, and the evolutionary conservation of the mutated position. Our results show that ELASPIC outperforms all other methods in predicting the effect of both core and interface mutations in high quality experimental datasets, achieving Pearson correlation coefficients of 0.77 for stability, and 0.75 for affinity predictions. Our analysis shows a major relevance of ΔΔG and ΔG_m_ together with molecular features describing volumes and overlaps, and evolutionary conservation for accurate prediction of stability changes. We also show that our predictor works in conjunction with modelled structures, thereby drastically increasing our coverage to a proteome-wide scale. While the prediction accuracy on modelled structures is lower, this will likely increase with better modeling techniques or further optimization of the structures using molecular dynamics or other related computationally expensive techniques.

Finally, we demonstrate that our predictor significantly distinguishes various types of disease-associated mutations obtained from Mendelian diseases, driver and passenger cancer mutations and common neutral ones. Interestingly, these results highlight the relevance of stability effects of mutations on domain folds and their interactions to the prediction of disease phenotypes. However, other relevant cellular factors, such as expression or protein essentiality, should be taken into account [55]. Therefore, ELASPIC should not be taken as a predictor of disease mutations, such as the sequence-based methods MutationTaster [56] or SNAP [57], since it was trained to predict stability rather than phenotype effect of mutations. However, we anticipate that the integration of physicochemical-based stability effects with genome wide information has the potential to provide more accurate predictions of disease phenotypes and better knowledge about the cause of the diseases at molecular level.

## Materials and Methods

### Experimental Datasets

#### Core mutations

To predict the stability effects of mutations in protein cores, we trained our core method on ProTherm [12], a thermodynamics database of protein and mutant stabilities. The list of proteins was curated at 90% identity using CD-HIT [58] in order to avoid biases in the training set due to over representation of some complexes. If multiple measurements were available, we selected values in kcal/mol near physiological conditions. Our modified ProTherm dataset used for training consists of 3,463 mutations in 159 proteins with crystal structures. To evaluate the performance of our core methods against other existing methods, we could not use our modified dataset, since these methods are not available for re-calculating them with a new training set. Alternatively, we used two benchmark datasets derived from old versions of ProTherm [12] to evaluate our core method against Popmusic2.0 [24], ProMaya [31] and Prethermut [44]. The Dehouck_09 [24] dataset consists of 2,636 mutations in 134 proteins, and Potapov_09 [19] dataset consists of 2,104 mutations in 79 proteins. To avoid redundancy, Potapov_09 selected the mean of multiple measurements, whereas Dehouck_09 employed a weighting scheme to prioritize mutations that were measured in or near physiological conditions.

#### Interface mutations

Our interface method has been trained and evaluated using the SKEMPI [13] database, in which experimentally measured stability changes of protein complexes upon amino acid mutation are available. To obtain an unbiased training set for our interface predictive model, the protein-protein complexes of SKEMPI were clustered using CD-HIT [58] with an average 90% sequence identity cut-off by combining both chains. From each cluster, the complex with the most available mutations was taken. To identify mutations at the interface, a 5 Å cut-off distance was applied between mutant atoms and the opposite chain. Our modified SKEMPI dataset consists of 873 mutations in 54 protein-protein complexes with crystal structures. Our interface method is evaluated against BeAtMuSiC [36] using ours and their own dataset, in which multiple mutations and redundant entries are removed from SKEMPI [13]. We could gather 2,027 mutations, which is comparable to the 2,007 obtained from BeAtMuSiC.

#### Disease mutations

In order to evaluate ELASPIC performance in identifying significant differences between neutral and disease-related mutations, we created three mutation categories with distinct stability signatures: OMIM, COSMIC and HapMap. Online Mendelian Inheritance in Man (OMIM [59]) mutations are those that cause Mendelian diseases, rare highly penetrating diseases with severe phenotypic consequences. We downloaded 18,635 OMIM mutations from Swissprot and were able to map 10,071 of them to a structure. Cancer on the other hand is caused by abnormal perturbations in cell regulation due to somatic mutations that result in uncontrolled cell proliferation and tumor formation [60]. Such changes are caused by “driver” mutations, i.e., mutations that provide a growth advantage. By contrast, the majority of somatic mutations in cancer are “passenger” mutations that accumulate in the cancer genome as a result of a breakdown of DNA repair processes [61]. To define driver and passenger mutations, we used cancer mutation frequency information from the Catalogue of Somatic Mutations in Cancer (COSMIC [62], [63]). Somatic missense mutations from 725,412 amino acid sites were downloaded from COSMIC (version 67). For our analysis, we classified driver mutations based on their occurrence in multiple independent tumor samples, whereas passenger mutations were present in single tumor samples [64]. Missense mutations were defined as a driver mutation if at least 5 distinct COSMIC samples from at least 3 distinct studies. To prevent bias from low throughput targeted gene analysis, we also selected as driver mutations those coming from at least 3 distinct genome screening based studies. Due to the computation resources needed to process these many mutations, we prioritized the calculations in decreasing order of reported mutation frequencies, completing 15,951 mutations. Finally, we obtained mutations with an expected neutral effect from HapMap, a catalog of common genetic variants that occur in human beings. We downloaded 9,141 neutral HapMap nsSNPs from BioMart and were able to map 1,206 to a structure.

### Generation of stability predictive features

Our integrative algorithm includes three categories of predictive features:

#### Sequence features

This category comprises domain length, BLOSUM80 and SIFT scores that are directly calculated for a given mutation in a protein. SIFT [16] software is used to predict the degree of sequence conservation for each mutated amino acid position and distinguish between deleterious from tolerated mutations.

#### Energy features

These are generated from existing structures, and in their absence, domains and complexes are structurally modeled. All mutated domains and complexes in both, core and interface datasets, have the wild-type structure associated to an entry in the Protein Data Bank, and experimental measurements for both, wild type and mutant variant. The first step to extract the structural features was to generate the wild type structure by isolating the PDB chain/s listed in the dataset, and the PDB ligands, defined as all HETATM excluding water molecules (i.e ATP, Ca^2+^). An empirical potential method, FoldX [23], was used to generate the mutant variant by inserting the single point mutations to the wild type structure using its standard procedure. FoldX was also used to perform a quick optimization in both wild type and mutant structures using a probability-based rotamer library to explore alternative conformations of the surrounding side chains. We used FoldX to generate descriptive energetic features derived from the calculation of stability and binding energies for the wild type and mutant variants, including the energy difference (ΔΔG). The stability energy was calculated using the *Stability* function from FoldX on the core datasets, whereas the binding energy was calculated using its *AnalyseComplex* function on the interface dataset. When using structures from homology modeling, we included additional features that describe the modelling accuracy: Modeller DOPE score and sequence identity between template and target sequences.

#### Molecular features

A description of the mutated position and its surrounding is obtained by several features. First, we calculate the number of intra- and inter- contacts between the residue of interest and the surrounding in order to describe the volumetric and physicochemical differences. Second, we classify atom contacts as hydrophobic (carbon-carbon), hydrophilic (hydrogen bond donor-acceptor) or electrostatic (positive-negative charged atom combinations). A distance cut-off of 5 Å and 4 Å were set for hydrophobic and hydrophilic/electrostatic interactions, respectively. For protein complexes, the solvent accessible surface area (SASA) of the interface and the hydrophobic, and hydrophilic contributions were calculated by subtracting the total SASA from the sum of the individual protomer SASA values using POPS software [65].

In total, we collected a list of 75 descriptors based on empirical, molecular and sequence features (Table S1)

### Identification and structural modelling of core mutations

To calculate the stability effects of disease-related mutations, we first have to identify the mutations that fall in a protein domain. For this, HMMER [66] software was used for searching Hidden Markov Model (HMM) profiles from Pfam families (v.27) [67] against the human sequences in Uniprot [68]. Once the family was identified, the final sequence boundaries for each predicted domain were extended using as a template the structure with the highest sequence identity. The template was identified by iterative pair-wise sequence alignments of the target protein sequence against all family members using Clustal Omega [69], a sequence alignment software. We distinguished core from surface mutations by obtaining the solvent inaccessible residues on the template structure, and mapping these positions to a target Uniprot domain, using the previous target-template sequence alignment. POPS software [65] was used to identify the core residues of the template structure, defined as those residues having SASA/Å^2^ of all side-chain atoms below 10 using a probe radius of 1.4 Å. For those mutations happening at the core of the target domain, we calculated the structural and energetic features. If an experimental structure for the exact target protein was not available (as is the case for the vast majority of proteins), we modelled the structure of domains based on the previous template structure using the software suite Modeller (v. 9.11) [70]. An accurate structure-based sequence alignment of target-template was generated with T-Coffee in "Expresso" mode, and modified to include HETATMs and to be compatible with Modeller. For each modelling run, 5 structures and 3 rounds of loop refinement were generated. Each structure was checked for knots with the KNOT [71] software to identify and eliminate aberrant models. Finally, the structure with the lowest normalized DOPE score (Modeller quality score) was selected for further free energy calculation.

### Identification and structural modelling of interface mutations

We distinguished surface from interface mutations by combining a structural classification of domain complexes with a protein-protein interaction network. First, we used physical interactions from Biogrid (v 3.2.95) [72] to obtain all UniProt protein-protein interaction pairs. For each protein pair, we obtained the domain composition for both proteins and checked whether there were interacting domain pairs of known structure in the PDB. Based on the assumption that interactions are conserved at the family level [39], we used SCOWLP [73] structural database of protein complexes to extract the different binding regions for a given domain-domain interaction. For each binding region, we obtained the best structural template, defined as the member with highest average sequence similarity per pair according to T-coffee, and with the highest structural resolution. The alignment was used to map the interfacial residues defined by SCOWLP to the template structure. For those disease-related mutations located at the interface, the domain-domain complex was modeled in the same way as for single domains (see previous section). Finally, the structural model of the complex was used to extract the different structure-based features as explained above (see Methods: Generation of stability predictive features).

### Predictive model

We treat the stability prediction problem as an instance of the non-linear regression problem. Our model assumes as input a vector of 75 derived features (Table S1), some of which are only applicable for interface mutations and hence are ignored for core mutations. We use the Stochastic Gradient Boosting of Decision Trees (SGB-DT) algorithm to fit a non-linear function to minimize the prediction error. SGB-DT belongs to the popular larger family of ensemble methods, which are based on the idea of using several weak predictors to build a powerful predictor. In our case, the weak learners are decision trees because they can model any non-linear functional dependencies without the need for complex pre-processing of data, i.e. transformations and normalizations. Ensemble methods differ in the way they combine the weak predictors http://en.wikipedia.org/wiki/Ensemble_learning. For example, http://en.wikipedia.org/wiki/Decision_treeboosting algorithms build the model in iterations of “boosting steps”, which update algorithm-specific weights on data points at each iteration by giving increasing focus to bad predictions. In particular, Gradient Tree Boosting fits a decision tree at each iteration on the residual errors of the ensemble model learnt up to the current iteration. The tree is fit on a subsample of the training set drawn at random without replacement, analogous to Random Forests, thereby leading to stochastic training. It then updates the full model by adding the newly learnt decision tree to the ensemble of trees (Refer to the work by Friedman [74] for a brief description of the algorithm). This provides scalability advantages as well as the so-called regularization effect that prevents over-fitting. Lastly, similar to Random Forest, SGB-DT has a built in feature importance measurement functionality, which allows end users to explore the predictive features in more details and understand the mechanics better.

### Training and testing with experimental structures

We trained and tested our method on the ProTherm (for core) and SKEMPI (for interface) databases independently. We took special precautions to prevent overlap and redundancy between the training and testing datasets (see above). Proteins and complexes in these datasets have experimentally solved crystal structures, thus no modeling is needed. We used the SGDRegressor implementation of the scikit-learn [75] machine learning toolkit to optimize parameters using cross validations. The parameters and the search space over which optimization is performed is given in Table S2. We used a 20-fold cross validation scheme with 50 repetitions in order to obtain a robust estimate of the mean and standard deviation of the achieved explained variance, i.e. Pearson correlation. This procedure was independently repeated for each dataset. Within each dataset, the cross validation scheme is used to split the dataset into training and test subsets to ensure non-overlapping training and test sets. The redundancy elimination in the pre-processing step (see above) removes dataset specific redundancies.

### Training and testing with modelled structures

In order to predict stability and affinity of mutations using modelled structures, we trained a separate predictor. Specifically, we discarded the experimental structures of the proteins in ProTherm and SKEMPI and instead built modelled structures. This was necessary as many features differed greatly between modelled and experimental structures; while this may reflect an inherent weakness in current homology modeling approaches, we sidestepped the issue by building a separate predictor to use for modelled structures. We hence modelled the sequences of the proteins and complexes for each mutation in the datasets. All possible template structures with resolutions ≤2 Å were taken independently of the sequence identities. All features were extracted as explained above in the modelling sections of the Methods. Those models presenting ΔG_wt_ values above 30 kcal/mol, and a normalized DOPE above 1 were not accepted for training. This cut-off was selected based on the maximum ΔG_wt_ folding/binding values observed for the subset of models done using templates at 100% sequence identity. These cases represent sequences modeled against their own experimental structure, where systematic biases of modelling affect the features. Our final modeled ProTherm dataset is composed of 4,449 mutations. From those, 22% have 100% seqID (sequence identity), 64% have 90% seqID or higher, and 14% have less than 50% seqID. Our modeled SKEMPI dataset is composed of 2,061 mutations. From those, 79% have 100% seqID (average sequence identity of both chains); 81% have 90% seqID or higher, 4% have less than 50% seqID. Unlike for the experimental dataset, we used a leave-one-family-out cross validation scheme in order to optimize hyper-parameters and measure model prediction performance. Each domain was associated to its family using Pfam database. The reason is to avoid family biases for those complexes with many structural templates at various identity levels. Mutations within these complexes would otherwise be represented by multiple instances in the dataset. Conversely, mutations without homologs in the PDB repository will be represented only once in the dataset modeled at 100% sequence identity. Therefore, our modelling strategy is also creating a family bias in terms of diversity. To cope with the redundancy of mutations and the sparseness of family-wise diversity, we utilized cross-validation at family level. At each fold of cross validation, we left out all mutations from a given family as test set, and built the regression model on the members of the remaining mutations. All hyper-parameters were optimized using the grid search as described in the previous section.

### Prediction of diseases mutations

Once the core and interface SGB-DT regression models were trained on the modeled (modified) datasets, we predicted the stability effects of the different disease mutation datasets to test the performance of our method on a larger scale. First, we identified the mutations located at the core of domains and at the interface of complexes. Then, we modelled the domains and complex structures to extract the energetic and molecular features together with the sequence features. Only significant models according to our previous definitions were selected for analysis. Finally, using the regression model we predicted the ΔΔG_DT_ (the predicted ΔΔG using machine learning) for each mutation. Results for each disease-related mutation dataset were analyzed using R software [76].

## Supporting Information

Figure S1 **Correlation between predicted and experimental ΔΔG values for our modelled version of the ProTherm core dataset (A) and SKEMPI interface dataset.**
(TIF)

Figure S2 **Correlation between predicted and experimental ΔΔG values for our modeled version of the SKEMPI interface dataset.**
(TIF)

Figure S3 **Box plots representing the distribution of the values for the most relevant features in core mutations.** Comparison is done for ProTherm experimental structures (orange), ProTherm modeled structures (red) and diseases mutations (yellow) in domain cores of proteins.
(TIF)

Figure S4 **Box plots representing the distribution of the values for the most relevant features in the interface mutations.** Comparison is done among SKEMPI experimental structures (orange), SKEMPI modeled structures (red) and diseases mutations (yellow) in protein interfaces.
(TIF)

Table S1 **Description of predictive features.**
(XLSX)

Table S2 **Hyper-parameters and their ranges, over which Stochastic Gradient Boosted Decision Trees (SGB-DT) algorithm is optimized.**
(XLS)

Table S3 **Feature importance for the training set on modeled structures for ProTherm and SKEMPI datasets.**
(XLSX)

Table S4 **P-values calculated with Wilcoxon Rank Sum and Kolmogorov–Smirnov test for comparison of distributions of stability predictions across various categories of mutations.**
(XLSX)
