# Supplementary figures and images for: Combining Structural Modeling with Ensemble Machine Learning to Accurately Predict Protein Fold Stability and Binding Affinity Effects upon Mutation
Source: PLoS One. 2014 Sep 22;9(9):e107353. doi: 10.1371/journal.pone.0107353 (PMC4170975; doi:10.1371/journal.pone.0107353)

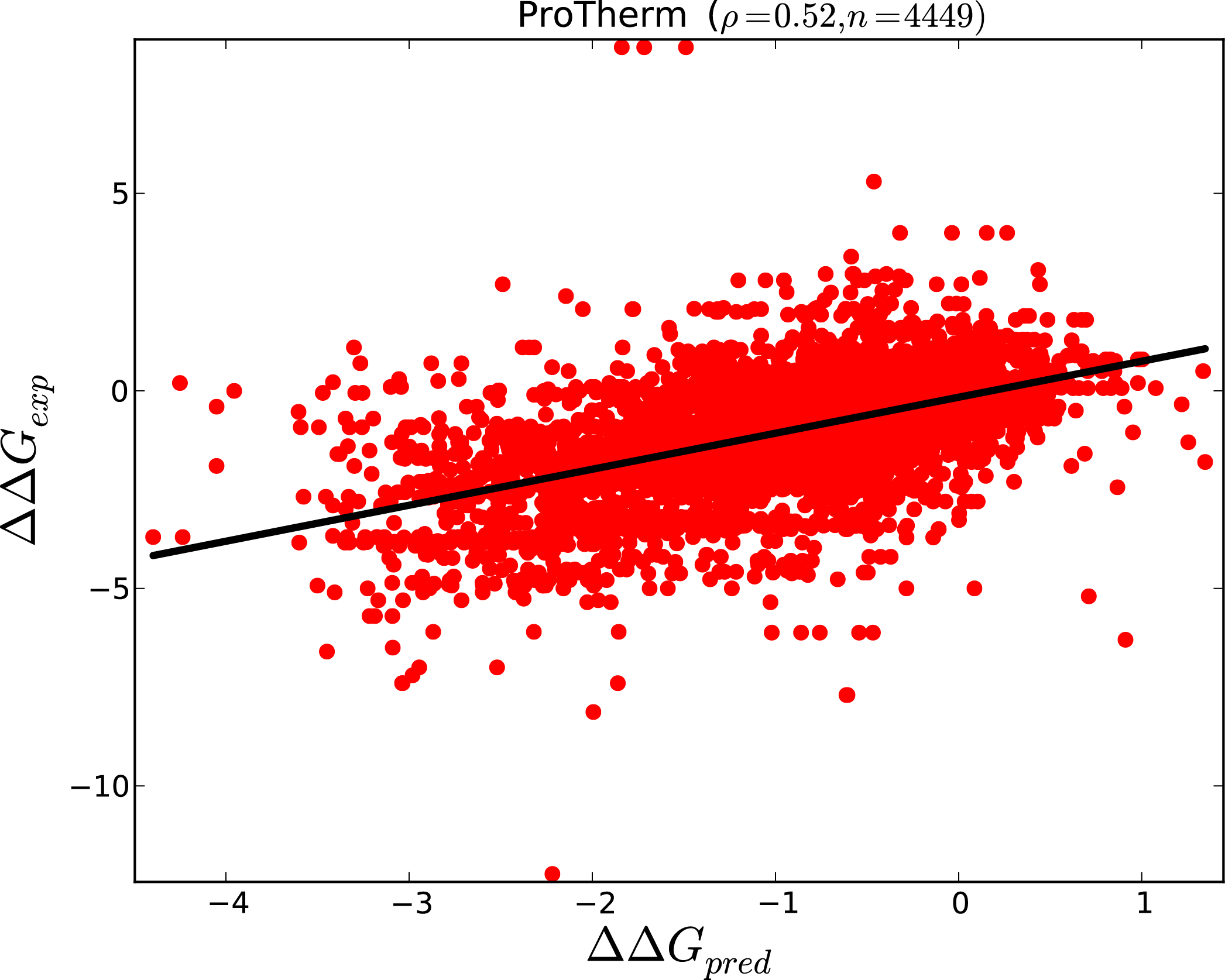

Supplement: Figure S1 — Correlation between predicted and experimental ΔΔG values for our modelled version of the ProTherm core dataset (A) and SKEMPI interface dataset. (TIF) [file pone.0107353.s001.tif]

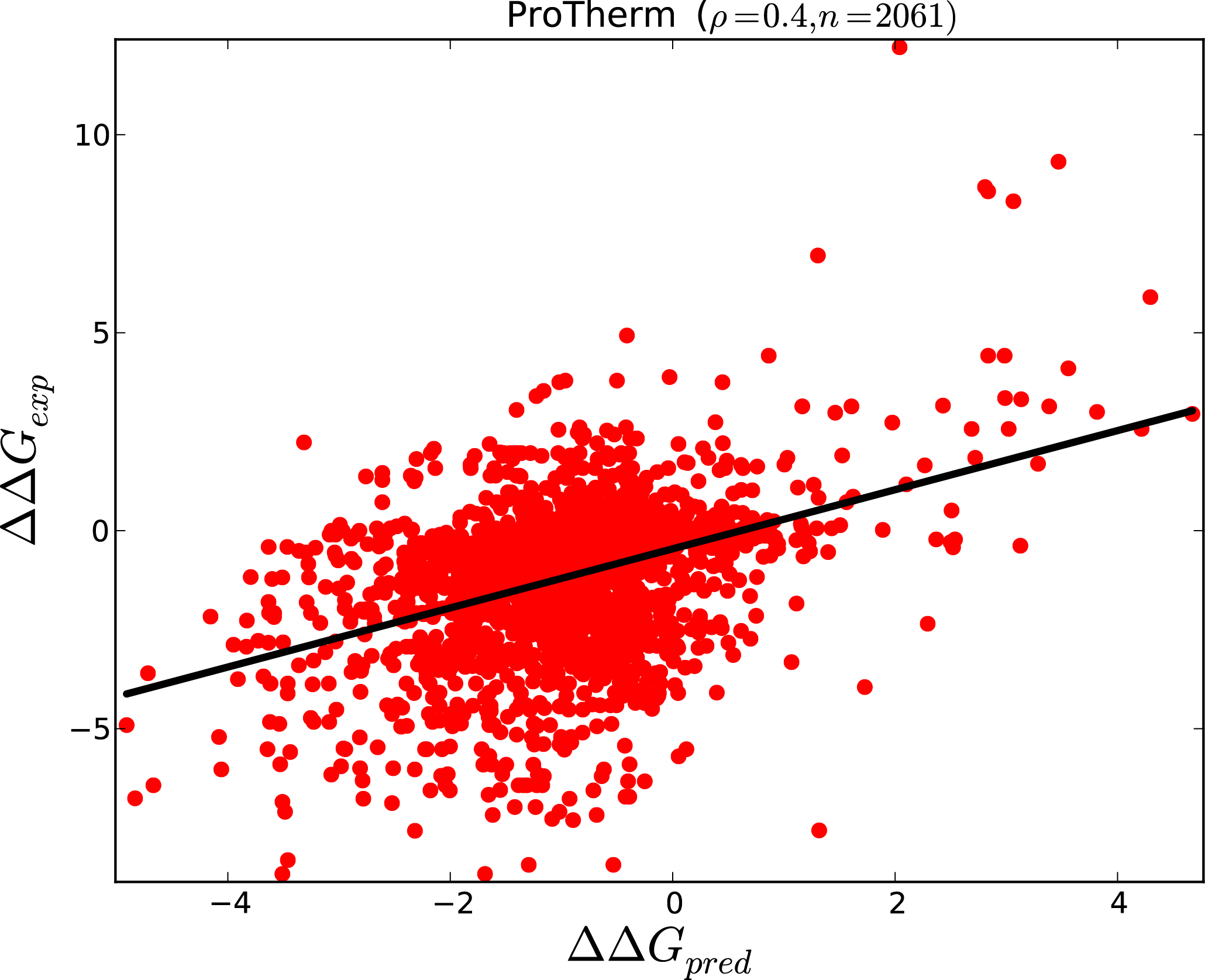

Supplement: Figure S2 — Correlation between predicted and experimental ΔΔG values for our modeled version of the SKEMPI interface dataset. (TIF) [file pone.0107353.s002.tif]

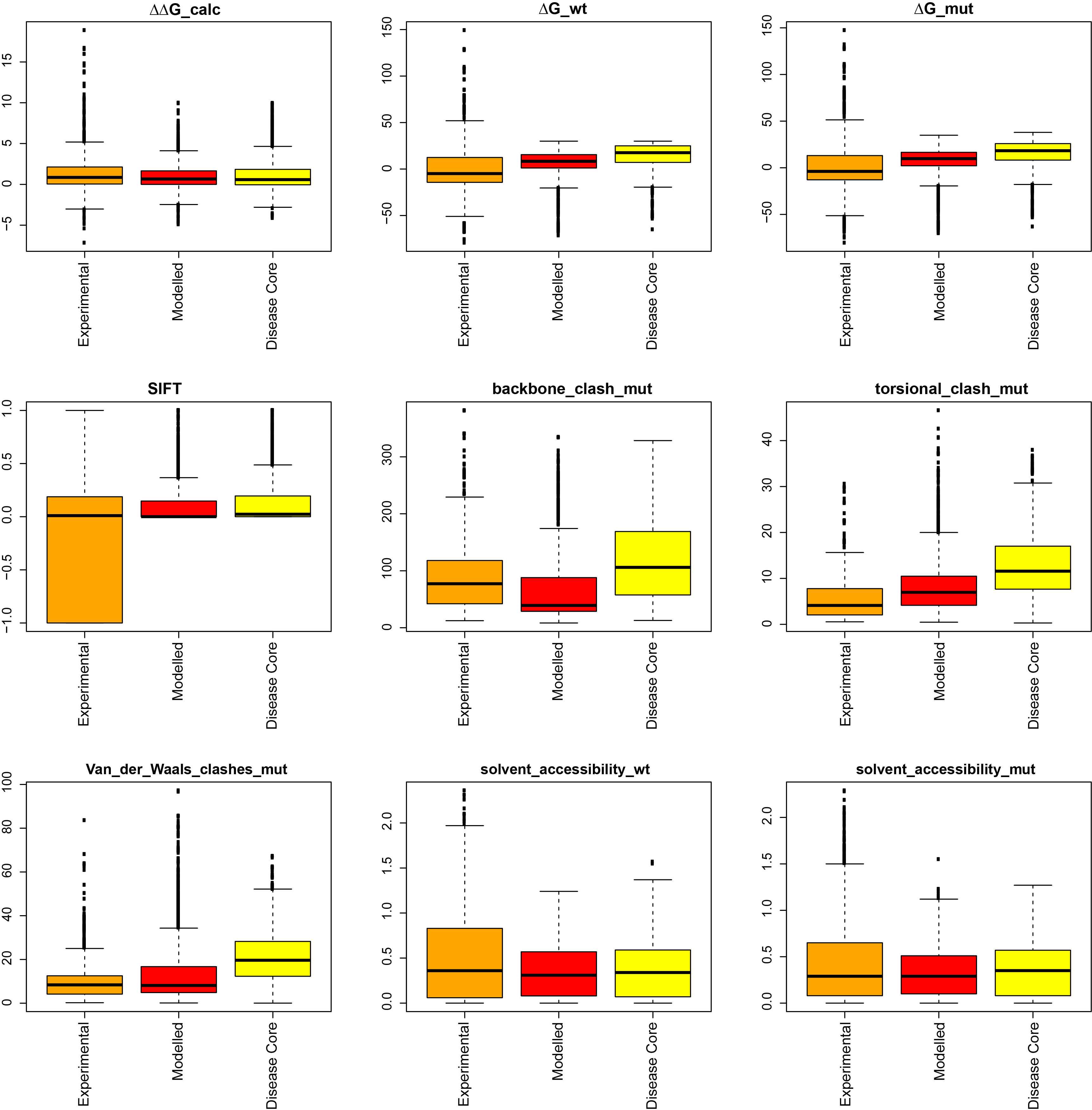

Supplement: Figure S3 — Box plots representing the distribution of the values for the most relevant features in core mutations. Comparison is done for ProTherm experimental structures (orange), ProTherm modeled structures (red) and diseases mutations (yellow) in domain cores of proteins. (TIF) [file pone.0107353.s003.tif]

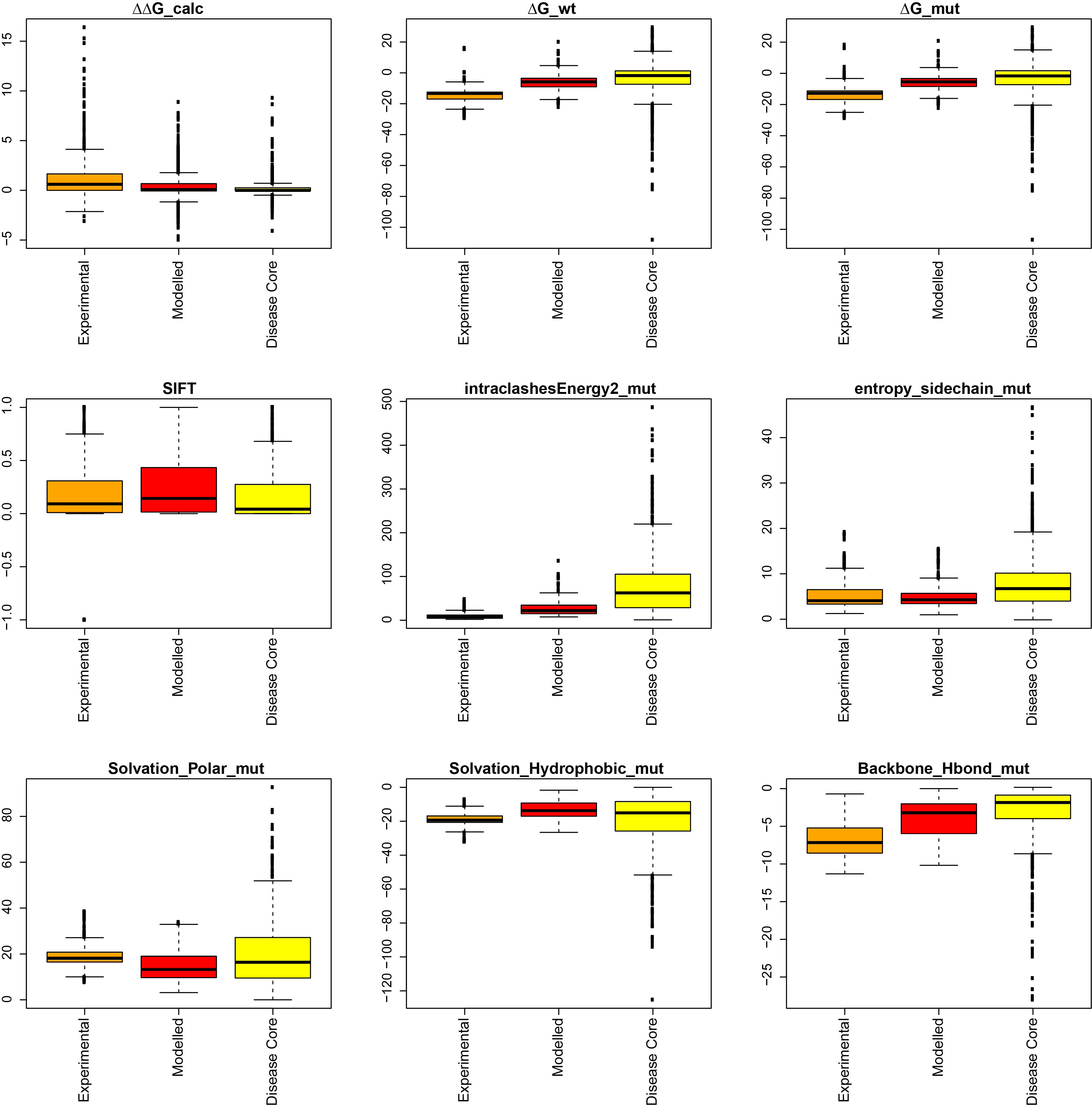

Supplement: Figure S4 — Box plots representing the distribution of the values for the most relevant features in the interface mutations. Comparison is done among SKEMPI experimental structures (orange), SKEMPI modeled structures (red) and diseases mutations (yellow) in protein interfaces. (TIF) [file pone.0107353.s004.tif]
